# Proteomic analyses of male contributions to honey bee sperm storage and mating

**DOI:** 10.1111/j.1365-2583.2006.00674.x

**Published:** 2006-10-01

**Authors:** A M Collins, T J Caperna, V Williams, W M Garrett, J D Evans

**Affiliations:** Bee Research Laboratory, ARS, USDA Beltsville, MD, USA; * Growth Biology Laboratory, ARS, USDA Beltsville, MD, USA; † Biotechnology and Germplasm Laboratory, ARS, USDA Beltsville, MD, USA

**Keywords:** seminal vesicles, social insect, semen, reproduction

## Abstract

Honey bee (*Apis mellifera* L.) queens mate early in life and store sperm for years. Male bees likely contribute significantly to sperm survival. Proteins were extracted from seminal vesicles and semen of mature drones, separated by electrophoresis, and analysed by peptide mass fingerprinting. Computer searches against three databases, general species, honey bees and fruit flies, were performed. Spectra were used to query the recently generated honey bee genome protein list as well as general species and fruit fly databases. Of the 69 unique honey bee proteins found, 66 are also in *Drosophila melanogaster*. Two proteins only matched honey bee genes and one is a widespread protein lost from the fly genome. There is over-representation of genes implicated in the glycolysis pathway. Metabolism-associated proteins were found primarily in the seminal vesicle. Male accessory gland proteins as identified in *Drosophila* rarely had orthologs among proteins found in the honey bee. A complete listing of gel spots chosen including honey bee genome matches and Mascot searches of MALDI-TOF results with statistics is in the Supplementary table. MALDI-TOF spectra and more complete Mascot peptide mass fingerprinting data are available on request. Supplementary figs 1–3 show the stained protein gels.

## Introduction

Honey bee (*Apis mellifera* L.) queens, as in most other social Hymenoptera, mate early in life and then store sperm to be used throughout their lifetimes. There has been long-standing interest in the mechanisms that allow social insect sperm to survive at ambient temperatures for years and even decades (Taber & Blum, 1960; Boomsma *et al*., 2005). For the honey bee, these mechanisms have a practical application with respect to the long-term preservation of semen for artificial insemination (AI) (Laidlaw, 1977; Harbo, 1985; Moritz, 1989). Given restrictions on the movement of honey bee genetic stocks, mandates to select bees resistant to various parasites and pathogens, and new tools available for defining genetic traits, germplasm preservation is a critical requirement for applied work in honey bees.

Our results from antioxidant enzyme activity (Weirich *et al*., 2002) and gene expression (Collins *et al*., 2004) assays and the remarkable durability of semen stored alone at room temperature (Collins, 2000b) indicated that the honey bee males (drones) contribute significantly to the physiology of sperm storage. Therefore we initiated the study reported here to identify male-produced proteins involved in honey bee mating and sperm storage.

Extensive studies for *Drosophila* and several other insects show that the products of male accessory glands have significant effects on female physiology and behaviour (reviewed by Chapman, 2001; Wolfner, 2002; Gillott, 2003; Kubli, 2003), as well as on successful sperm transport and storage (Neubaum & Wolfner, 1999). Eighty-three accessory gland proteins (Acps) have been identified from flies (Wolfner, 2002) some of which have been confirmed to stimulate egg production and laying, reduce female receptivity to mates, mediate sperm storage, and provide microbial protection in the reproductive tract. Both seminal fluid and sperm must be present for the full range of effects to be expressed. We carried out computational searches for honey bee orthologs to the described fly Acps in this project with poor success, suggesting that the known high rates of sequence divergence for many of these proteins might make them indistinguishable across the 300 million years (my) separating flies and bees. Accordingly, we then carried out *de novo* searches for honey bee proteins implicated in sperm storage, by proteomic (MALDI-TOF peptide mass fingerprinting) analyses of proteins present in the seminal vesicles and in semen itself.

**Figure 1:**
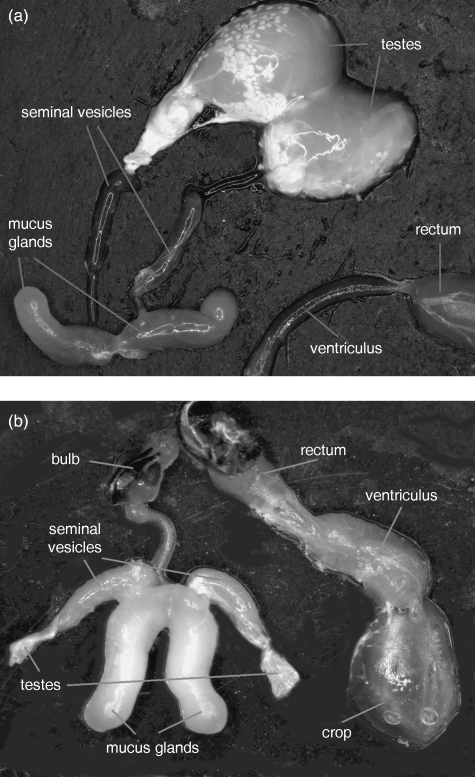
The reproductive tracts of an immature (a) and mature (b) honey bee drone. Testes, seminal vesicles and mucus glands are labelled.

By the time that adult drones emerge from pupation spermatogenesis and spermiogenesis are complete (Bishop, 1920; Hoage & Kessel, 1968). During the first week of adult life, the sperm migrate from the testes to the seminal vesicles (Snodgrass, 1963) where they undergo the final stages of maturation. Figure 1 shows the dramatic difference in size of the testes of immature (Fig. 1a) and mature (Fig. 1b) drones. Also note that the seminal vesicles and the mucus glands become filled in the mature drone. The epithelial cells lining the seminal vesicles secrete a small amount of seminal fluid during the maturation, making up about half of the total semen volume (Verma & Shuel, 1973). At the same time mucus is being produced and stored in the mucus glands.

At the time of mating, muscles in the abdomen of the drone contract creating pressure that everts the genitalia into the queen, turning the reproductive system inside out (Koeniger, 1986). The muscular contractions of the seminal vesicles aid in the ejaculation of the semen into the queen's vaginal passage and on to the median and lateral oviducts. The products of the male mucus gland (Colonello & Hartfelder, 2003) follow the semen and harden on contact to air (Bishop, 1920; AC and VW, personal observation). Immediately after eversion, the male is paralysed and falls away from the queen. The mucus plus the endophallus and cornua portions of the male genitalia, which are left behind, become the mating sign, visible outside of the queen's abdomen. From comparisons of mating behaviour in several species of *Apis* (Koeniger, 1990; Koeniger & Koeniger, 2000) it appears that the mucus and genital structures hold the drone in place during sperm transfer and the mating sign becomes a signal to other males following the queen. Koeniger (1986) proposed that the mucus may also serve to hold the queen's sting out of the way of the next mate.

The honey bee queen makes one to four flights (Roberts, 1944) away from the colony when she is about a week old, mating with from seven to 44 males in rapid succession (Taber, 1954; Moritz *et al*., 1996; Neumann *et al*., 1999; Tarpy & Page, 2001). Over the next 24–48 h, the sperm migrate to the spermatheca (Ruttner *et al.*, 1971). This movement is enabled by contractions in the queen's abdomen (Koeniger, 1986), by sperm motility (Collins, 2000a) and the presence of spermathecal fluid (Gessner & Ruttner, 1977). Only about 3–5% of the sperm are actually retained in the spermatheca (Koeniger & Koeniger, 2000), the rest are lost from the queen.

Genetic studies using phenotypic markers have shown that sperm from all of the mated drones become randomly distributed within the spermatheca (Page *et al*., 1984) and relatively constant levels of all paternal types are represented in the worker offspring. Therefore there is little or no sperm competition, although different drones may contribute variable numbers of viable sperm (Woyke & Jasinski, 1978; Collins, 2004). The creation of colonies of multiple paternal lines of workers, or subfamilies, is evolutionarily desirable, as these colonies have the capability of responding readily to wide changes in the environment (Jones *et al*., 2004) and extreme polyandry increases the fitness of the queen by reducing the colony-level impact of her laying non-viable, diploid drone eggs (Tarpy & Page, 2001).

## Results

A total of 234 spots were cut from 2-dimensional gels and analysed using peptide mass fingerprinting (MALDI-TOF) against the Genbank nunredundant (nr) and official bee protein sets, 90 from seminal vesicle tissue and 144 from semen, 90 picked from one gel (pH 3–10) and 54 from a second gel (pH 7–10). Using Mascot and the bee-specific database, we found significant GLEAN3 matches from 60 protein spots derived from seminal vesicles (54 unique protein IDs), plus one additional match to a novel peptide predicted by Genscan. We found 78 GLEAN3 matches to genes present in honey bee semen (33 of these are unique). Because we ran gels with overlapping pH ranges for the separation of semen proteins, there was greater redundancy of spots from these samples. There was also some biological redundancy with several neighbouring spots showing the same database matches. Tissues and semen were collected from six source drones and it is possible that redundant spots reflect both allelic (isozymic) variation as well as variation in the production or post-translational processing of proteins. In support of the latter, many identical matches were derived from spots with significantly different size or pH traits (Table 3). Interestingly we found no spots that matched non-bee entries in the GenBank database that did not also match a honey bee gene, attesting to both the completeness of the bee gene list and the difficulty in making Mascot fingerprinting identifications over this long evolutionary distance. A complete listing of all spots and results of analyses are listed in the Supplementary table.

**Table 3:** Official bee protein matches that were found in multiple spots

Bee gene	No.	Spot no.
GB10485	2	A33, B38
GB10973	2	A46, B55
GB11965	2	A7, A8
GB14798	2	A49, C35
GB15079	2	C8, C9
GB15550	2	B43, B44
GB16546	2	B32, C20
GB17626	2	A26, B26
GB18917	2	A77, A78
GB19387	2	A39, B39
GB12546	3	B40, B41, B42
GB13058	3	A15, B85, C3
GB14311	3	A14, B8, B88
GB18293	3	A54, B61, B62
GB14501	4	B16, B17, C5, C26
GB15039	4	A34, B27, B28, C17
GB15561	4	A68, B56, C36, C37
GB17473	4	B72, B73, B82, C49
GB10514	5	A18, A19, B10, B11, B13
GB14018	5	A57, A81, B63, B64, B86
GB10122	6	B12, B21, B22, B23, C7, C10
GB17864	6	A35, A36, B34, B36, C27, C28
GB18109	7	A51, A52, A53, B57, B58, B59, C38
BGB15171	B12	A24, A60, A72, B30, B31, B60
		B67, B69, C16, C18, C43, C45
BGB16905	B13	A21, A22, B18, B19, B20, B33
		B74, C6, C11, C13, C21, C23, C24
BGB15463	B15	A44, A45, A68, B43, B44, B45, B46
		B47, B48, B56, C30, C31, C32, C36, C37
From mixed samples		
GB18538	3	A3, A4, A5

Of the 69 unique honey bee proteins found 66 showed significant BLASTP matches to proteins in the genome of *Drosophila melanogaster* (*e*-value < 1.0 × e^−10^, Tables 1 and 2). Two of the remaining proteins (GB11987, spot A13; GB13059, spots A15, B85, C3; and GB18752, spots B90, C44) showed no non-bee matches in nr while the third *de novo* predicted gene (Group 1.17.1, spot A47) seems to be a widespread protein lost from the fly genome.

**Table 1:** Proteins predicted from seminal vesicle tissue that matched a honey bee gene. Gene ontology biological functions inferred via *Drosophila* matches

Gene name	Tissue	Dros42match	Dros42name	Dros42eval	GOGroup	Biological function (first)
GB10275	sem ves	CG9277	betaTub56D		0 Cyt	cytoskeleton organization and biogenesis
GB11920	sem ves	CG3401	betaTub60D		0 Cyt	axon guidance
GB13049	sem ves	CG9277	betaTub56D		0 Cyt	cytoskeleton organization and biogenesis
GB14141	sem ves	CG3937	cher		0 Cyt	cytoskeleton organization and biogenesis
GB18365	sem ves	CG18290	Act87E		0 Cyt	cytoskeleton organization and biogenesis
GB92659	sem ves	CG8938	GstS1		8.1E-37 Cyt	cytoskeleton organization and biogenesis
GB10133	sem ves	CG11793	Sod		2.2E-57 DE	ageing
GB10498	sem ves	CG11765	Prx2540		1.4E-95 DE	defensemen responsemen
GB12741	sem ves	CG3752	Aldh		0 DE	defensemen responsemen
GB14852	sem ves	CG4264	Hsc70		0 DE	defensemen responsemen
GB19860	sem ves	CG8542	Hsc70		0 DE	defensemen responsemen
GB10989	sem ves	CG3762	Vha68		0 Enrg	ATP biosynthesis
GB11385	sem ves	CG3731	CG3731		0 Enrg	mitochondrial electron transport
GB13596	sem ves	CG11154	ATPsyn		0 Enrg	ATP biosynthesis
GB19171	sem ves	CG17369	Vha55		0 Enrg	ATP biosynthesis
GB92538	sem ves	CG7460	CG7460		3.5E-33 Enrg	electron transport
GB19460	sem ves	CG6058	Ald		5E-144 GLY	glycolysis
GB10139	sem ves	CG1743	Gs2		1E-160 Met	glutamate catabolism
GB15662	sem ves	CG10962	CG10962		2.4E-44 Met	metabolism
GB16448	sem ves	CG5730	AnnIX		1E-123 Met	lipid metabolism
GB17238	sem ves	CG11876	CG11876		2E-138 Met	pyruvate metabolism
GB19030	sem ves	CG10638	CG10638		5E-96 Met	aldehyde metabolism
GB10710	sem ves	CG5939	Prm		0 Mus	mesoderm development
GB10939	sem ves	CG4843	Tm2		2E-121 Mus	muscle contraction
GB11965	sem ves	CG17927	Mhc		0 Mus	cytokinesis
GB13399	sem ves	CG2184	Mlc2		4E-55 Mus	muscle contraction
GB18538	sem ves	CG5939	Prm		5E-159 Mus	mesoderm development
GB18917	sem ves	CG4254	tsr		2.1E-73 Mus	actin filament depolymerization
Group1-17.1	sem ves	CG4898	Tm1		3.6E-33 Mus	dendrite morphogenesis
GB11987	sem ves	none	n/a	n/a	n/a	n/a
GB13058	sem ves	none	n/a	n/a	n/a	n/a
GB16951	sem ves	CG10120	Men		0 TCA	tricarboxylic acid cycle
GB17439	sem ves	CG17246	Scs		0 TCA	tricarboxylic acid cycle
GB15718	sem ves	CG14207	CG14207		1.1E-68	none
GB18647	sem ves	CG10691	l(2)37Cc		2E-116	DNA replication
GB18969	sem ves	CG12101	Hsp60		0	de novo′ protein folding

GO group assigned by 3 biological function notations. Only first is cited in table.

**Table 2:** Proteins predicted from semen alone or from semen and seminal vesicle tissue that matched a honey bee gene. Gene ontology biological functions inferred via *Drosophila* matches

Gene name	tissue	Dros42match	Dros42name	Dros42eval	GOGroup	Biological function (first)
GB10485	both	CG3085	CG3085		1E-113 Cyt	microtubule cytoskeleton
GB14018	both	CG6647	porin		7.5E-32 Enrg	anion transport
GB14798	both	CG12055	Gapdh1		1E-143 GLY	glycolysis
GB15039	both	CG17654	Eno		2E-135 GLY	glycolysis
GB15052	both	CG1721	Pglym78		6E-113 GLY	glycolysis
GB15463	both	CG6058	Ald		1.9E-97 GLY	glycolysis
GB17626	both	CG7430	CG7430		0 GLY	glycolysis
GB19387	both	CG3001	Hex		9E-158 GLY	glycolysis
GB15561	both	CG10160	ImpL3		1.8E-91 GLYOX	glyoxylate cycle
GB15171	both	CG8782	Oat		4E-85 Met	amino acid biosynthesis
GB16738	both	CG7113	scu		7.7E-52 Met	acyl-CoA metabolism
GB16905	both	CG7920	CG7920		3E-141 Met	acetyl-CoA metabolism
GB17864	both	CG7920	CG7920		6E-155 Met	acetyl-CoA metabolism
GB18293	both	CG11876	CG11876		2E-111 Met	pyruvate metabolism
GB10514	both	CG1913	alphaTub84B		0	cell motility
GB10973	both	CG32031	Argk		2E-168	phosphorylation
GB14311	both	CG3213	CG3213		2.4E-34	protein biosynthesis
GB18109	both	CG6084	CG6084		3E-117	n/a
GB10122	semen	CG9277	betaTub56D		0 Cyt	cytoskeleton organization and biogenesis
GB14501	semen	CG32819	CG32819		1E-160 Cyt	microtubule cytoskeleton
GB11769	semen	CG17146	Adk1		6.3E-46 Enrg	ATP metabolism
GB11056	semen	CG3127	Pgk		1E-173 GLY	glycolysis
GB16546	semen	CG7430	CG7430		6E-158 GLY	glycolysis
GB17473	semen	CG2171	Tpi		3E-80 GLY	glycolysis
GB14517	semen	CG7176	Idh		0 GLYOX	glyoxylate cycle
GB10467	semen	CG4233	Got2		0 Met	aspartate metabolism
GB12546	semen	CG7899	Acph		2.4E-53 Met	phosphate metabolism
GB15079	semen	CG5320	Gdh		0 Met	NOT sperm storage
GB18752	semen	none	n/a	n/a	n/a	n/a
GB11512	semen	CG7264	CG7264		4.1E-36	n/a
GB12716	semen	CG4409	CG4409		2.8E-11	n/a
GB14399	semen	CG18335	CG18335		0	n/a
GB15550	semen	CG32031	Argk		2E-119	phosphorylation

Eighteen identified proteins were found in both seminal vesicle and semen samples, 36 were unique to the seminal vesicle and only 15 were unique to semen (although they might have come from somewhere else in the male reproductive tract). This latter number is an under-representation of semen-unique proteins by Fisher's exact test (*P* = 0.04) (Sokal & Rohlf, 1995). Proteins were grouped by Gene Ontology biological processes using the parent terms cytoskeleton, defense, energy, glycolysis, glyoxylate cycle, metabolism, muscle, tricarboxylic acid cycle or other (Tables 1 and 2). The identified proteins from this sampling were diverse (37 of 69 had distinct GO biological functions) but included some apparent functional biases. Overall, the proteins showed an abundance of genes involved in metabolism and cytoskeletal function. The GO parent groupings fell out differentially for the two protein sources (Table 4; global *G*-test, *P* = 0.003), with an excess of muscle-related and cytoskeletal genes in the seminal-vesicle proteins and an excess of proteins related to glycolysis (and possibly the glyoxylate cycle) in the semen samples. Most proteins (38 of 66 unique GB matches) were predicted by pSortII to reside in the cytoplasm, followed by mitochondria (18 of 66). Only two proteins (GB12716 and GB18752) were predicted to be extracellular, and both of these were found only in the semen component.

**Table 4:** Protein matches grouped into Gene Ontology biological function classes

GO Class	Seminal vesicle only	Semen only	Both	Total
Cytoskeleton	6	2	1	9
Defense	5	0	0	5
Glycolysis	1	3	6	10
Glyoxylate cycle	0	1	1	2
Metabolism	5	3	5	13
Mitochondrial/energy	5	1	1	7
Muscle	7	0	0	7
Other	3	4	4	11
TCA	2	0	0	2
Total	34	14	18	66

## Discussion

Sperm is stored by honey bee queens, and queens of other social insects, for years prior to fertilization. In honey bees, sperm has also been shown to remain viable for extended periods *in vitro* at ambient temperatures. Collins (2000a) held semen alone in sealed capillary tubes up to 1 year, and found viability did not begin to drop until about 9 weeks and in some samples remained above 50% for up to 6 months, a remarkable survival rate compared with other higher animals.

This survey provides the first global look at sperm-related proteins in honey bees and the first, to our knowledge, genome-enabled proteomic survey of semen proteins in an insect. The results indicate constitutional biases in sperm-related proteins as well as information on the potential sources of semen proteins. A high number of the proteins identified in these samples showed significant matches to *Drosophila* (66 of 69 at < e^−10^) indicating that this is a fairly conserved set of proteins.

When organized into GO groups there is an apparent over-representation of genes implicated in the glycolysis pathway (10 of 55 proteins that could be associated with a GO biological function). Other carbohydrate metabolism genes were also present (from the Krebs’ and glyoxylate cycles) along with two antioxidative enzymes, superoxide dismutase (SOD), and glutathione-S-transferase (GOT). Regarding SOD and GOT, previous studies of enzyme activity (Weirich *et al*., 2002) and gene expression (Collins *et al*., 2004) targeted these as two of three potential antioxidants in reproductive tissues. SOD was expressed in semen, one-third of the activity directly associated with the sperm fraction, and at similar levels in the spermathecae of mated and virgin queens. GOT was found in the spermatheca at higher levels after mating, but not in semen. Our third subject enzyme in these previous studies, catalase, was significant in that it was almost entirely associated with the sperm itself, as opposed to seminal fluid and was also expressed at higher levels in spermathecae of mated queens (Weirich *et al*., 2002). It is surprising that we did not identify catalase in the current survey, a result perhaps reflective of its low abundance in seminal fluid and/or its membrane association in sperm.

We found significant biases in metabolism-associated proteins in semen vs. the seminal vesicle. Blum *et al*. (1962) found high levels of sugars, especially glucose, fructose and trehalose, in the seminal fluid as well as phospholipids. The sugars were rapidly oxidized. Verma & Shuel (1973; Verma, 1978) speculated that the sugars and phospholipids were the primary sources of energy for motility of sperm. Previous observations of motility in honey bee semen by Poole & Edwards (1970) demonstrated that the spermatozoa became non-motile within a short period, but the addition of sugars to the semen restored motility. Verma & Shuel, (1973) also found that sperm were motile for only 6 h in an extender with no oxidizable substrate. While we can not directly infer the metabolic processes endogenous to sperm (and seminal fluid) from this project, the protein biases are suggestive of the specific metabolic (and catabolic) activities that sustain sperm prior to and during mating.

A comparative study of mammalian, avian and honey bee semen by Kraft *et al*. (1978) reported that honey bee sperm were unusual in that they used only the Embden–Meyerhof glycolysis cycle for metabolism, and not the Krebs’ cycle. They commented that this would be highly adaptive for sperm that remained inactive for extended periods. We found no TCA enzymes in semen, although there were two in the seminal vesicles.

The Acps from flies rarely have orthologs in bees based on the honey bee genome annotation (HBGSC, 2006). This may reflect shorter Acp length, but it seems more likely that this is a corroboration of the dogma of fast-evolution for these genes. Mueller *et al*. (2004) used comparative structural modelling to infer conserved protein functional classes of 52 of the predicted seminal proteins. These classes included: regulators of proteolysis, lipid-modifying enzymes (lipase-related), lectins, cysteine-rich proteins of the CRISP family, an antioxidant, antimicrobial peptides, and one RNase. In our continuing studies of the reproductive tissues of the honey bee, we will look for proteins of similar function. The proteolytic proteins may be involved in sperm motility, and similar Acps in *Drosophila* may serve as coagulation factors related to mating plug formation. Proteins with lipid-binding sites and lectins would participate in lipid and carbohydrate metabolism, respectively, already suggested as the energy sources for honey bee sperm (Verma & Shuel, 1973). The presence of an antioxidant agrees with our own studies showing three such enzymes as important components of semen and present at high levels in the spermatheca. We did not include mucus glands in the present study, but Colonello & Hartfelder (2003) reported only three dominant and three minor polypeptides present.

High rates of sequence evolution aside, it is not too surprising that a group of proteins intimately associated with reproductive biology are dissimilar between two insects with such different reproductive behaviour and physiology. The *Drosophila* female generally mates with one male at a time, although she may mate again after oviposition. There is behavioural competition between male *Drosophila* for mates during extensive courtship rituals. The matings generally last for 15–20 min, also excluding other males, and there is documented sperm competition for fertilization (Simmons, 2001). Proteins from the male accessory gland in seminal fluid and the mating plug aid in protection of the male investment by reducing the female tendency to mate again, and spur the initiation of egg laying. For the honey bee drone the key competitive hurdle is in reaching the female first from the drone ‘comet’ that follows her in flight – a competition that relies more on visual acuity and flight physiology. Matings are rapid, taking a matter of seconds, and females often mate several times in immediate succession (Koeniger, 1986). In contrast to many insects (Gillott, 2003) the honey bee mating plug does not seem to negatively affect the success of subsequent mates, and indeed might actually aid this success (Koeniger, 1986). Nor does it affect the future viability of the queen (Colonello & Hartfelder, 2005). While mating barriers, *per se*, seem less likely in honey bees than in other insects, the possibility still remains that the drone-provided proteins have strong effects on the physiology of queens.

*Drosophila* males have one pair of accessory glands with two cell types (Chapman & Davis, 2004); drones have two separate glands, paired seminal vesicles and mucus glands. Similar research on the mucus gland of the honey bee shows that by maturity the number of components has been reduced to three dominant polypeptides and three minor ones (Colonello & Hartfelder, 2003). For the bumble bee, *Bombus terrestris*, Baer *et al*. (2000, 2001) report that the mucus plug contains four fatty acids and a dipeptide, but it is the fatty acid, linoleic acid, which inhibits remating. This species mates only once. The mucus/genitalia-tissue plug of the stingless bee, *Melipona quadrifasciata*, inhibits remating also, but simply by the mechanical stimulation of the queen's abdomen (Melo *et al*., 2001).

We know little or nothing about changes that take place in the spermatozoa as they move from the testes to the seminal vesicles to the spermatheca to fertilization of the egg. In other domestic animals, primarily mammals, there are significant changes (activation, capacitation and the acrosome reaction) that occur during the migration within the male and after deposition in the female reproductive tract that are critical to the final act of fertilization of the egg. However, there have been few indications of similar shifts in physiology during sperm maturation or sperm storage in insects. The proteins found in the seminal vesicle and semen might guide us to a better understanding of sperm developmental stages in the honey bee and thus to insects in general.

Similar proteomic and genomic-centred analyses continue with a focus on the spermatheca and its contents, before and after mating. Differences in pH between the semen and reproductive tract that function for orientation of the sperm to the spermathecal duct, may be only one factor in the changed metabolic processes during storage. Future studies also need to investigate enzymes in testes, where spermiogenesis occurs, as well as the ejaculatory and spermathecal ducts. In a histochemical study Schoeters & Billen (2000) found polysaccharides in spermathecal ducts of queens but not workers, and suggested that these sugars provided energy for activation of sperm prior to fertilization. Klenk *et al*. (2004) identified an SDS–PAGE gel band from spermathecal fluid as a 29 kDa protein somewhat homologous to triosephosphate isomerase. They suggested that it was not serving a glycolytic function in the queen but aiding in sperm storage. Results from our continuing studies will provide more insight into the remarkable durability of honey bee sperm and how it is supported by the queen's physiology.

The work reported here highlights the invaluable data that have been made available through the Honey Bee Genome Project. Our initial peptide mass fingerprinting efforts using existing databases for honey bees and other insects showed < 5% hit rates, a 10-fold lower rate than when the honey bee genomic data were included. The availability of a genome-wide predicted bee protein list and the genome sequences themselves was essential to this project and will provide extensive new opportunities for proteomic research in honey bees.

## Experimental procedures

### Chemicals

Chemicals for electrophoresis including acrylamide, bis-acrylamide, SDS, TEMED, ammonium persulphate, agarose, 2-mercaptoethanol, CHAPS, Triton-X-100 and ampholytes (Bio-Lytes, pH 3–10 and pH 7–10) were purchased from Bio-Rad (Hercules, CA, USA). Trizma-Base (Tris), EDTA, dithiothreitol (DTT), glycerol and thiourea, were purchased from Sigma (St Louis, MO, USA). Alpha-cyanohydroxycinnamic acid (CHCA) matrix was purchased from Bruker Daltonics (Billerica, MA, USA) and urea was from Pierce (Sequanal grade, Rockford, IL, USA). All other chemicals were reagent or HPLC grade laboratory chemicals.

### Tissue collection

Free-flying mature drones of commercial Italian stock were collected from several colonies maintained by the Bee Research Laboratory, Beltsville, MD, USA. Drones that had overwintered in the hive in Maryland were collected in February. Seminal vesicles were removed from six drones, those that did not evert in the process of dissection, and were frozen at −80 °C in a polypropylene tube. Semen was collected in November using the standard method of ejaculation and collection in a sterile capillary tube (Harbo, 1974). The 28 µl pooled sample representing 34 drones was also stored frozen at −80 °C in a polypropylene tube.

### Protein extraction from tissues

Proteins were extracted from tissues essentially using the method described by Stadler & Hales (2002). Briefly, tissues were homogenized mechanically with a pestle in 0.15 ml 10 mm sodium phosphate buffer in 139 mm sodium chloride, pH 7.2. One millilitre of cold chloroform/methanol (1 : 4), was added, vortexed and sonicated. The solution was centrifuged at 16 000 ***g*** for 10 min at 4 °C. Supernatant solution was discarded and methanol (1 ml) was added to the pellet, which was mechanically disrupted, sonicated and centrifuged as before. The final pellet was vacuum dried to remove residual methanol and re-suspended in IPG strip rehydration buffer: 8 m urea, 1 m thiourea, 50 mm DTT, 4% CHAPS, 0.5% Bio-Lytes using mechanical disruption and sonication. Insoluble material was removed by centrifugation at 15 000 *g* for 2 min at room temperature. The concentration of protein in tissue extracts was determined by the method of Lowry (Peterson, 1977) following precipitation with TCA (Nerurkar *et al*., 1981). Bovine serum albumin was used as a standard.

### Two-dimensional polyacrylamide gel electrophoresis (2D-PAGE)

The first dimension IEF was performed using 11 cm IPG strips (pH 3–10 or pH 7–10, Bio-Rad) in the IPGphor II system (GE Healthcare, Piscataway, NJ, USA). All IPG strips were rehydrated in the presence of 500 µg protein in a total volume 185 µl rehydration buffer; additional rehydration buffer contained the following: 8 m urea, 4% CHAPS, 50 mm DTT, 0.5% Bio-Lytes, 0.001% bromophenol blue. The strips were passively rehydrated for 16 h at 20 °C and voltage settings for isoelectric focusing were: 500 V for 30 min, 1000 V for 30 min, and 8000 V maximum to a total of 30 kVh. The focused strips were either immediately run on a second dimension polyacrylamide gel or rinsed in water and then stored at −80 °C. For the second dimension gel electrophoresis, the gel strips were incubated with equilibration buffer 1 (0.4 m Tris–HCl pH 8.8, 6 m urea, 20% glycerol, 2% SDS, 1% DTT, 0.001% bromophenol blue) for 5 min and fresh buffer was added for an additional 15 min. Strips were then incubated in the same equilibration buffer containing 2.5% iodoacetamide for an additional 15 min. Finally, excess iodoacetamide was removed by soaking strips (5 min) in the buffer containing 0.06 m Tris–HCl (pH 6.7), 6 m urea, 20% glycerol, 2% SDS, 0.001% bromophenol blue, 0.5% DTT. Each strip was rinsed in dH20 and subsequently placed on to a 12% polyacrylamide gel (16 × 20 cm) with Tris–glycine buffer system as described by Laemmli (1970). Strips were overlayed with 2.5% low melting agarose sealing solution (0.06 m Tris base, 0.1% SDS, 0.01 m DTT and 0.001% bromophenol blue, pH 6.7). Electrophoresis was performed overnight, using the PROTEAN II xi system (Bio-Rad) at 30 °C using a 2 × buffer solution in the top chamber (0.38 m glycine, 0.5 m Tris, 0.2% SDS, pH 8.3) and a 1 × buffer solution in the bottom chamber for 1100–1200 Vh and 9.5 mA/gel. The 2-D PAGE gels were visualized by staining with Colloidal Coomassie Blue G-250 (Newsholme *et al*., 2000). Briefly, gels were fixed overnight in 50% ethanol and 3% phosphoric acid followed by three washes with distilled water. Gels were prestained for 1 h in 34% methanol, 17% ammonium sulphate and 3% phosphoric acid and finally the gels were stained in the same solution containing Coomassie Blue G-250 (0.066%) for 2–3 days. The gels were stored in 10% ammonium sulphate solution and scanned using laser densitometry (PDSI, GE Healthcare). Duplicate gels were used for each protein sample.

### In gel digestion of proteins

Protein spots were excised from the stained gel and stored in dH20. Thawed gel material was washed first with a destaining solution water/methanol/acetic acid (4.5 : 4.5 : 1), then in dH20 and finally in 50% acetonitrile containing 25 mm ammonium bicarbonate. The gel plug was dehydrated with 100% acetonitrile, dried under vacuum, and then re-swollen with 20 µl of 10 µg/ml trypsin (modified porcine trypsin, sequencing grade, Promega, Madison, WI, USA) in 25 mm ammonium bicarbonate. An additional 20 µl 25 mm ammonium bicarbonate buffer was added to cover the gel plug. Digestion was performed overnight at 37 °C. The resulting tryptic fragments were extracted with 50% acetonitrile and 5% trifluoroacetic acid (TFA) with sonication. The extract was dried to completeness by vacuum centrifugation and then dissolved in 50% acetonitrile and 0.1% TFA.

### Protein identification

#### MALDI-TOF-MS analysis

A Voyager DE-STR MALDI-TOF mass spectrometer (Applied Biosystems, Framingham, MA, USA) operated in positive ion reflector mode was used to analyse tryptic peptides. Samples were cocrystallized with α-cyanohydroxycinnamic acid (CHCA) matrix, and spectra were acquired with 50 shots of a 337 nm Nitrogen Laser operating at 20 Hz. Spectra were calibrated using the trypsin autolysis peaks at m/z 842.51 and 2211.10 as internal standards.

#### Database searching

Protein identification was performed by searching two distinct databases. First, the US National Center for Biotechnology Information (NCBI) nonredundant database was searched using the Mascot search engine on the Matrix Science public domain server (http://www.matrixscience.com). Second, a honey bee specific database was generated using all of the genome-annotated protein calls (GLEAN3, HBGSC, 2006) combined with a partially redundant database of *ab initio* proteins predicted by Genscan (Burge & Karlin, 1997) and FGENESH+ (http://www.softberry.com). In total this database contained 46 720 protein sequences and 14 133 680 residues specific to the honey bee genome. Peptide mass fingerprints were searched against this database on a local Mascot server. The following parameters were used for both nr and local database searches with peptide mass fingerprinting data: monoisotopic mass, 25 p.p.m. mass accuracy, trypsin as digesting enzyme with one missed cleavage allowed and carbamidomethylation of cysteine, oxidation of methionine, N-terminal pyroglutamic acid from glutamic acid or glutamine as allowable variable modifications. Taxonomy was limited to animals for the searches of NCBI nr database, no taxonomy limitations were imposed on the searches of the bee database as it contained only *Apis mellifera* sequences. To qualify the MALDI-TOF-MS data as a positive identification, a protein's score was equal to or exceeded the minimum significant score. For searches of the NCBI nr database, that cut-off score for a significant (*P* < 0.05) match was 72, and for the bee database 59. In the end, there were no matches to nr database that were not reflected as matches against the honey bee-specific database. Accordingly, all sequence comparisons were carried out using the honey bee match set.

### Comparative sequence analysis

Unique honey bee proteins identified through Mascot (*n* = 70) were used in local BLASTP searches against the *Drosophila melanogaster* genome sequence (version 4.2, http://www.flybase.org), with a significance cut-off of 1.0 × e^−5^ and the BLOSUM62 search algorithm. Honey bee proteins that failed to find a *Drosophila* match were then used to query the NCBI nr database (at http://www.ncbi.nlm.nih.gov) by BLASTP and the same conditions. *Drosophila* matches to the honey bee proteins were placed on to chromosomal locations, and into Gene Ontology (GO) Biological Function child terms (database downloaded from http://www.godatabase.org/cgi-bin/amigo/go.cgi). These terms were then grouped, where possible into higher level GO biological function terms. Honey bee protein predictions were also placed into likely subcellular and extracellular locations using the trained sorting algorithm pSortII, as described at http://wolfsport.seq.cbrc.jp

### Statistical analyses of results

Honey bee protein matches were divided into those found only in seminal vesicle extractions, those found only in semen extractions, and those found in both extractions. *G*-tests were used to assess overall distributional bias between these tissues, and to survey for differential presence of specific GO parent terms in either of the two sample types.

## Supplementary material

Supplementary table

This material is available as part of the online article from http://www.blackwell-synergy.com
