# Supplementary material for: Proteomic analyses of male contributions to honey bee sperm storage and mating
Source: Insect Mol Biol. 2006 Oct 1;15(5):541–9. doi: 10.1111/j.1365-2583.2006.00674.x (PMC1847503; doi:10.1111/j.1365-2583.2006.00674.x)
Supplement: Figure S1 [file imb0015-0541-fs1.pdf]

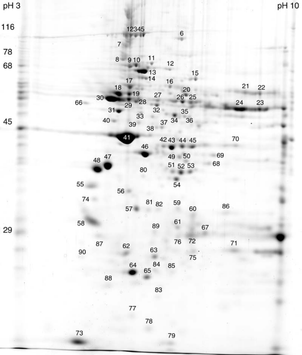
pH 3
pH 10
116
78
68
45
29
1 2 3 4 5
6
7
8
9
10
11
12
13
14
15
16
17
18
19
20
21
22
23
24
25
26
27
28
29
30
31
32
33
34
35
36
37
38
39
40
41
42
43
44
45
46
47
48
49
50
51
52
53
54
55
56
57
58
59
60
61
62
63
64
65
66
67
68
69
70
71
72
73
74
75
76
77
78
79
80
81
82
83
84
85
86
87
88
89
90
