# Supplementary figures and images for: Proteomic analyses of male contributions to honey bee sperm storage and mating
Source: Insect Mol Biol. 2006 Oct 1;15(5):541–9. doi: 10.1111/j.1365-2583.2006.00674.x (PMC1847503; doi:10.1111/j.1365-2583.2006.00674.x)

pH 3

pH 10

116

78

68

45

29

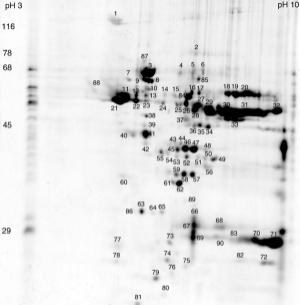

Supplement: Figure S2 [file imb0015-0541-fs2.pdf]
