# Supplementary material for: Proteomic analyses of male contributions to honey bee sperm storage and mating
Source: Insect Mol Biol. 2006 Oct 1;15(5):541–9. doi: 10.1111/j.1365-2583.2006.00674.x (PMC1847503; doi:10.1111/j.1365-2583.2006.00674.x)
Supplement: Figure S3 [file imb0015-0541-fs3.pdf]

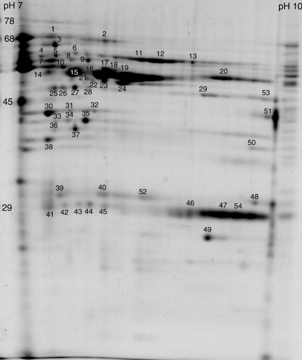
pH 7
pH 10
78
68
45
29
1 2 3 4 5 6 7 8 9 10 11 12 13 14 15 16 17 18 19 20 21 22 23 24 25 26 27 28 29 30 31 32 33 34 35 36 37 38 39 40 41 42 43 44 45 46 47 48 49 50 51 52 53 54
